# Dr. Michael H. Thaut, Ph.D., Professor of Music at the University of Toronto, Canada: Adapted Interview from the 12^th^ World Congress for NeuroRehabilitation (WCNR), Vienna, 2022

**DOI:** 10.25122/jml-2023-1014

**Published:** 2023-03

**Authors:** Stefana-Andrada Dobran, Alexandra Gherman

**Affiliations:** 1 RoNeuro Institute for Neurological Research and Diagnostic, Cluj-Napoca, Romania; 2 Sociology Department, Babes-Bolyai University, Cluj-Napoca, Romania

**Interviewer: Stefana-Andrada Dobran**

**Interviewee: Professor Michael H. Thaut**

Dr. Michael H. Thaut, Ph.D., is currently a Professor of Music at the University of Toronto with cross-appointments in the Faculty of Medicine [Medical Sciences, Rehabilitation Science and Neuroscience]. He also holds appointments as Collaborator Scientist at the CAMH Neuroimaging Research Center and as Affiliate Scientist at the Li Ka Shing Knowledge Institute at St. Michael’s Hospital, Toronto. He acts as Director of the Music and Health Science Research Center (MAHRC) and Music and Health Sciences Graduate programs at the University of Toronto. He is also endowed as TIER I Canada Research Chair.

Dr. Thaut is the past president of the International Society for Clinical Neuromusicology and Vice-President of the International Society for Music and Medicine.

Professor Michael Thaut is an international research leader in the neuroscience of music and the applications of auditory neuroscience to neurological rehabilitation. He has over 250 scientific publications and is the co-editor of the Oxford Handbook of Music Psychology, the first editor of the Oxford Handbook of Music and Brain Research, and the Oxford Handbook of Neurologic Music Therapy.

He and his team developed the clinical system of Neurologic Music Therapy (*the therapeutic use of music applied to sensory, speech and language, cognitive, and motor dysfunctions after a neurologic event or diagnosis*), which is applied worldwide in neurorehabilitation, and endorsed as evidence-based by the World Federation of Neurorehabilitation.

**S.A.D.:** Hello, dear Professor Thaut! We are here, in Vienna, for the 12^th^ World Congress for NeuroRehabilitation, organised by the World Federation for NeuroRehabilitation. What is your first-hand opinion of the event so far, and have you participated in any previous editions?

**M.T.:** I have been at these congresses presenting actively since 2006 in Hong Kong, so I’ve been to quite a few of those, and they were all great. This is a good congress; it’s exciting because it’s coming back from lockdowns and Zooms, so everybody is excited, and this is a great step to bring people back together, and Vienna is a great place to do this. There’s a lot of buzz and excitement going on right now; that’s my impression.

**S.A.D.:** What do you believe is the overarching theme of this year’s congress?

**M.T.:** The overarching theme is always the same – trying to help people with neurologic disorders. It’s a big variety in what you are looking at in terms of stroke and Parkinson’s, traumatic brain injury, and many other disorders, so the idea is - can we find ways to disseminate and bring neurological rehabilitation to everything in the world? Because it is not that evenly distributed concerning quality, quantity, availability, and accessibility, so I think this is one of the big missions of WFNR.

**S.A.D.:** From your perspective, what is the role of hybrid multidisciplinary events in developing neurorehabilitation research and practice, and what other avenues do you believe are worth exploring?

**M.T.:** The idea of ‘hybrid’ is important because there is an accessibility advantage when you can do this online, and so you are catching people coming back in person, which is great, but hybrid availability creates accessibility across the world; it just changes everything, so I think we will continue – in my judgment, we will probably continue with hybrid multidisciplinary events for quite a while. Or maybe never really get rid of it because you can reach people online – in a hybrid set-up that could not afford to come to a conference. So, it’s also – we have done a lot of research in the last two or three years, not by choice, where we had to use a kind of hybrid and online technology to collect data, so I think some of the things are good and the good ones, the good part will stay, and some things were sort of more artificially forced upon us and I would hope they are already starting to disappear. But the idea of ‘hybrid’ is actually an idea that I would support strongly because it just gives accessibility to this kind of knowledge, across the whole world, without people having to spend three, four, or five hours to travel.

**S.A.D.:** What is the impact of neurological music therapy (NMT) on the recovery of neurologic functions?

**M.T.:** Neurologic music therapy is something that we developed based on research on how the brain processes music. And as we researched that, we found that there are many aspects of brain processing in music or in auditory stimulation, which translate actually very well in rehabilitation set-ups. We know, for instance, that auditory rhythm perception translates into motor control improved in stroke and Parkinson’s. When they are synchronized, they walk into a steady beat. The role of NMT has really taken on a big curve, in visibility and in effectiveness in the last 20 years. And in the last 3 or 4 years, we also had again some major breakthroughs in terms of mechanisms, understanding how music-pace interventions actually create clinical change associated with brain plasticity. I think the role of NMTs (neurological music therapists), the way we see it, will continue strongly, and the Academy for Neurologic Music Therapy, which sort of guides the training and certification process, is now also an institutional member of WFNR.

**S.A.D.:** On the same topic, do you believe there is enough awareness of NMT usage for brain rehabilitation?

**M.T.:** I think the awareness has grown tremendously, but there is still a lot to do. It started as a very small, new development in terms of looking at music as a different kind of therapy, not a different music therapy, and I think there are about 4.000 certified NMT therapists in 65 countries, so it’s relatively a small number still, but it’s growing tremendously, and some of the NMT intervention techniques are actually in the standards of stroke care guidelines in the United States and Canada, so there is a big medical recognition step. In the United States, of the top 5 neurorehabilitation hospitals, 3 of them actually have NMT teams. There is growth, but I would say we are working hard to create more visibility and access to this form of therapy.

**S.A.D.:** Actually, this was my last question, if you believe there is enough access to this kind of therapy worldwide, or what can we do to improve access?

**M.T.:** It is still limited. The distribution is 4.000 across 65 countries. Then, some countries, like the U.K., Germany, the U.S., Canada, and the Netherlands, have maybe 300, 400, U.S. – maybe 1.000 NMTs, but if you spread this out across other countries, then the numbers get pretty small. Access to a broader audience is important, and I think the institutional membership of the NMT Academy now with the WFNR helps to spread NMT word and disseminate it in newer and much more comprehensive ways.

**S.A.D.:** Thank you very much for the interview!

**M.T.:** Thank you!

